# Distinguishing verrucous herpes from neoplastic lesions: A clinical and histopathological approach

**DOI:** 10.1016/j.jdcr.2026.02.051

**Published:** 2026-04-10

**Authors:** Mounika Vattigunta, Jose A. Jaller

**Affiliations:** Dr. Phillip Frost Department of Dermatology and Cutaneous Surgery, University of Miami Miller School of Medicine, Miami, Florida

**Keywords:** Herpes Simplex Virus, squamous cell carcinoma

## Case description

**Fig 1:**
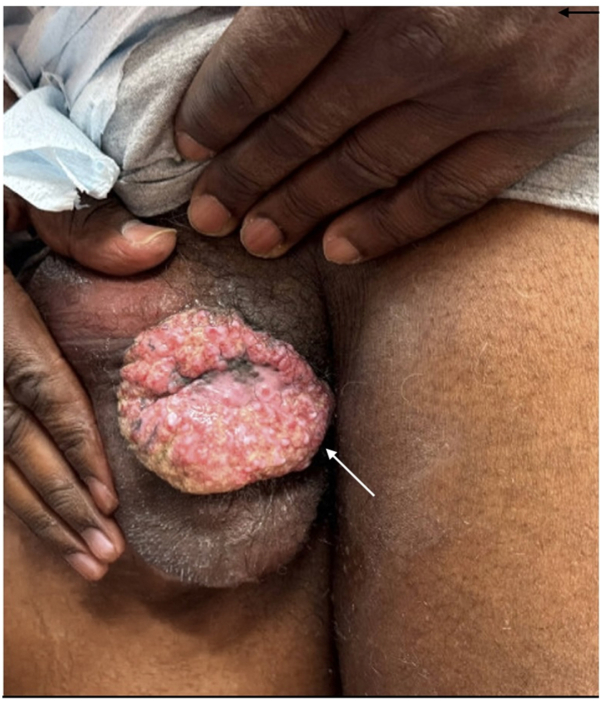
Verrucous herpes as a right scrotal mass. *Arrow* is pointing to a right scrotal mass.

**Fig 2:**
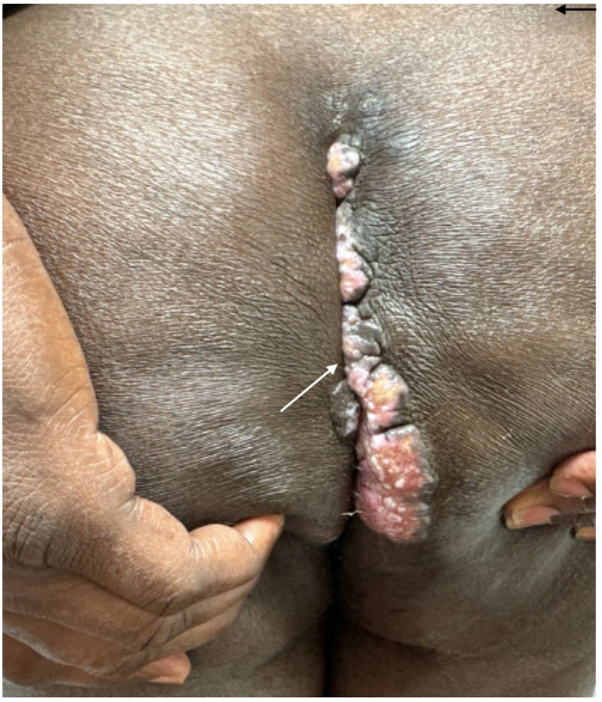
Verrucous herpes localized to gluteal cleft. *Arrow* is pointing to a right scrotal mass.

**Fig 3:**
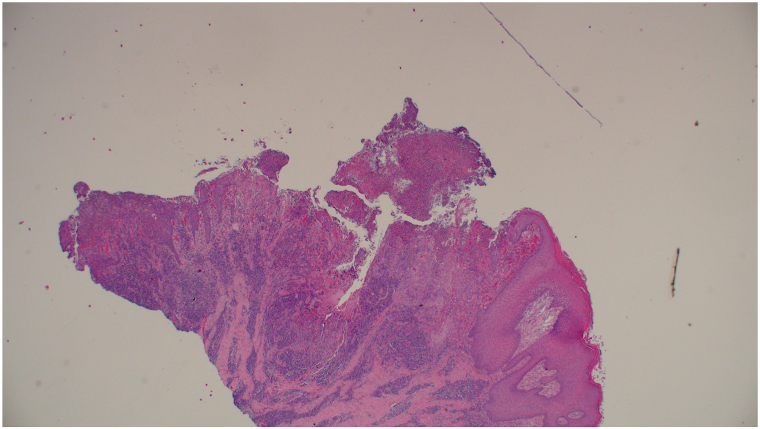
Representative histopathological image showing regular epidermal acanthosis with a prominent stratum lucidum. There is mild acute and chronic inflammation in the upper dermis with mild hyperkeratosis.

**Fig 4:**
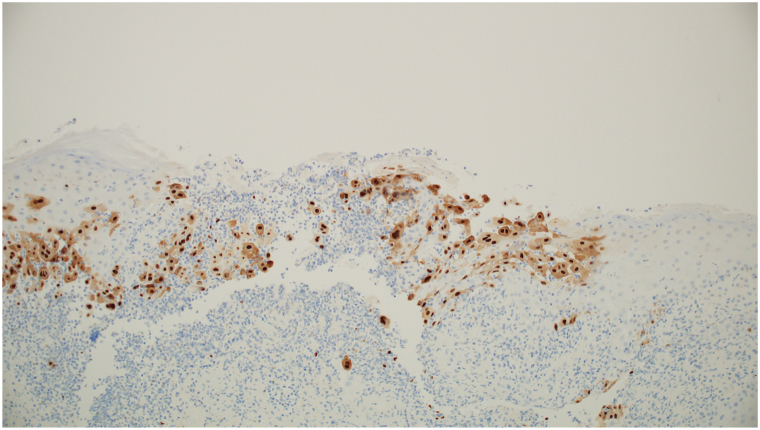
Immunohistochemistry positive for HSV antigen within epidermal keratinocytes.

A 62-year-old man with HIV (CD4 count: 233 cells/μL) presented to the dermatology clinic with a 3-month history of a draining mass on the right scrotum and gluteal cleft. Physical examination revealed an 8-cm eroded, vegetative, draining tumor on the right scrotum (Fig 1) and verrucous nodules along the gluteal cleft (Fig 2). Prior to biopsy, clinical differential included squamous cell carcinoma versus condyloma acuminata versus verrucous herpes simplex virus (HSV). Incisional biopsies demonstrated regular acanthosis with intraepidermal collections of acantholytic cells containing nuclear inclusion bodies (Fig 3). Immunoperoxidase studies were positive for HSV, confirming the diagnosis of HSV infection with associated epidermal acanthosis (Fig 4).

Verrucous HSV is an atypical presentation of HSV that typically occurs in immunocompromised patients, particularly those with HIV.^1^ It manifests as wart-like, ulcerative lesions that grow in clusters and can be misdiagnosed as condyloma acuminata or squamous cell carcinoma.

Treatment typically includes antiviral medications such as acyclovir, valacyclovir, and famciclovir, although some patients might need intralesional^2^ or surgical treatment.^3^ Patients in an immunocompromised state may not respond to standard anti-herpetic agents such as acyclovir, thus possibly needing escalation of treatment.^1^ This case highlights the importance of maintaining a broad differential diagnosis when evaluating cutaneous lesions in immunocompromised patients.

These lesions can be mistaken for a malignant mass. A diagnosis of squamous cell cancer caused by HPV should be high on the differential. Along with atypical morphology, the lesions present in atypical locations, which is seen in this case. Verrucous herpes occurs due to an exophytic, proliferative response to HSV in immunocompromised patients.

**Question: A 62-year-old man with HIV (CD4 count <250** **cells/mm) presents with a chronic, draining, vegetative tumor on the scrotum and gluteal cleft. Biopsy reveals intraepidermal acantholytic cells with nuclear inclusions. Which of the following is least likely to be true regarding this condition?**

**A.** This presentation represents an atypical exophytic proliferative response to HSV in immunocompromised hosts
**B.** It is often misdiagnosed clinically as HPV-induced condyloma acuminata or squamous cell carcinoma
**C.** Standard oral acyclovir is used as treatment as antiviral resistance is uncommon in immunocompromised patients
**D.** Escalated treatment options include intralesional cidofovir or systemic foscarnet
**E.** Histology typically shows pseudoepitheliomatous hyperplasia with multinucleated keratinocytes with opaque and ground glass nuclei

## Discussion

C is the correct answer as acyclovir-resistant HSV is more prevalent in immunocompromised hosts^4^ and thus treatment-resistance cases are often treated with intralesional cidofovir or systemic foscarnet. Vegetative HSV occurs most commonly in immunocompromised patients.^1^ The vegetative manner in which verrucous HSV presents often mimics condyloma acuminata or squamous cell carcinoma.^1^ Histologic examination of verrucous HSV includes pseudoepitheliomatous hyperplasia with multinucleated keratinocytes with opaque and ground glass nuclei (Fig 3).^5^

### Declaration of generative AI and AI-assisted technologies in the writing process

None.

## Conflicts of interest

None disclosed.
